# A generic and adaptable model of developing, implementing, and maintaining patient-reported outcomes in clinical practice – the AmbuFlex model

**DOI:** 10.1186/s41687-026-01076-3

**Published:** 2026-05-04

**Authors:** Liv Marit Valen Schougaard, Anne Jessen, Anne Treschow Phillipsen, Anke de Beijer, Camilla Buur Udbye Agesen, Helle Dahl Harbo, Jonas Hermann Schrøder, Kathrine Hønholt Carlson, Katja Kimose Sloth, Lene Bjerre Graakjaer, Louise Pape Larsen, Mette Roost, Nanna Toxvig Sørensen, Regine Grytnes, Sanne Degn Gjørup, Sidse Østergaard Olesen, Birgith Engelst Grove

**Affiliations:** 1 AmbuFlex, Centre for Patient-Reported Outcomes, Gødstrup Hospital, Hospitalsparken 15, Herning, 7400 Denmark; 2 Department of Clinical Medicine, Aarhus University, Palle Juul-Jensens Boulevard 82, Aarhus N, 8200 Denmark

**Keywords:** Patient reported outcome measures, Clinical practice, Implementation, Co-design

## Abstract

**Background:**

Patient-reported outcomes (PROs) are aspects of health status reported directly by patients. Over the last decade, PROs have been increasingly used in clinical practice. AmbuFlex – Centre for Patient Reported Outcomes is a public-funded organization that develops, implements, and maintains PRO solutions in the Danish healthcare system. Since 2011, a total of 90 specific AmbuFlex PRO solutions have been implemented across diverse patient groups and hospital departments. AmbuFlex’ implementation model is based on extensive real-life experience and inspired by theoretical frameworks, such as the Consolidated Framework for Implementation Research, the Medical Research Council’s framework of development and evaluation of complex interventions, and the PROTEUS Guide to Implementing PROs in Clinical Practice. In this paper, we describe the AmbuFlex model for developing, implementing, and maintaining PRO solutions in clinical practice.

**Results:**

The process outlined in the AmbuFlex model comprises five steps: (1) Clarify PRO rationale, (2) Select PROMs, (3) Design PRO solution, (4) Implement and adjust PRO solution, and (5) Monitor, evaluate and expand PRO solution. Throughout the process, the active involvement of relevant stakeholders is essential. A PRO facilitator manages this iterative process, and a series of meetings are held with a clinical working group based on specific contextualized requirements.

**Conclusion:**

The AmbuFlex model is a generic and adaptable model, that applies to the development, implementation, and maintenance of PRO solutions regardless of purpose, target group, clinical context, and stakeholders involved. Thus, the model is useful for developing and implementing sustainable PRO solutions in the healthcare system.

## Background

Patient-reported outcomes (PROs) are aspects of health status reported directly by patients using standardized questionnaires, also termed PRO measures (PROMs) [1]. In clinical practice, PROMs support screening and monitoring of the individual patient’s health status and promote patient-centered care [2]. PROMs have shown to improve patient-clinician communication and self-management [3–6]. When using PROMs remotely, one of the approaches is to triage the need for clinical attention during treatment or follow-up aiming to allocate resources to patients with the greatest needs [7–9]. This approach has shown lower use of healthcare services without compromising safety and quality of care in various chronic conditions [10–13]. However, implementation of PROMs in clinical practice is a complex process demanding a thorough implementation strategy. The existing strategies strongly vary, and so does the degree of successful implementations of PROMs in clinical practice [14]. Barriers that influence the implementation process relate to perceived low value by patients and healthcare providers, PROMs being too complex or difficult for patients to complete, poor usability of PRO systems, delayed feedback of PRO data, and resource constraints [15]. Facilitators include the presence of phased implementation, professional training, stakeholder engagement prior to implementation, clear strategies and goals, and ‘change champions’ to support implementation and integration into patient pathways [15].

AmbuFlex – Centre for Patient-reported Outcomes is a public-funded organization that facilitates the development, implementation, and maintenance of PRO solutions in two Danish regions [7, 8]. From 2011 to 2026, AmbuFlex has implemented 90 unique PRO solutions across diverse patient groups. The overall aim is to achieve patient-centered care and to optimize the use of resources in the healthcare system. The AmbuFlex organization consists of a team of PRO facilitators, an IT team, and a research team. The PRO facilitators develop, implement, and maintain PRO solutions in close collaboration with patients and clinicians. The IT team maintains and develops new features for a PRO system, ensuring functions that support the ongoing changes in clinical practice, and the research team conducts mixed-method research in the use of PROMs in clinical practice. Our implementation model is based on extensive real-life experience since 2011, and specific methods in our model have been revised over time. In addition, our model is inspired by theoretical frameworks, including the Consolidated Framework for Implementation Research (CFIR) 2.0 [16], the Medical Research Council’s (MRC) framework of development and evaluation of complex interventions [17], and the PROTEUS Guide to Implementing PROs in Clinical Practice [18]. The three frameworks are relevant to consider in the implementation of PRO solutions and complement each other.

The CFIR framework presents a variety of constructs organized into five domains: innovation, the outer setting, the inner setting, the individuals, and the implementation process [16]. The overall aim of the CFIR framework is to explain barriers and facilitators to implementation effectiveness, and it is commonly used in PRO implementation initiatives in the healthcare system [14, 19]. In AmbuFlex, ‘innovation’ is defined as a PRO solution that consists of three core elements: a PRO questionnaire/PROMs, supporting materials, (e.g., the clinical workflow), and an IT-system. A PRO solution depends on its potential users and can only bring value to clinical practice if it is used by both patients and clinicians (the individuals). Patients and clinicians are part of a hospital department (inner setting), and local readiness and resources will affect how the PRO solution functions in clinical practice. At the same time, the clinical use of a PRO solution will also affect the existing clinical workflows and the patient care pathway. This underscores the importance of engaging stakeholders and considering organizational factors throughout the entire implementation process. The hospital department is part of a larger organizational healthcare system (outer setting), and the political agenda affects the execution of innovative initiatives in the healthcare system.

A PRO solution is a complex intervention as it targets a change in behavior among patients and clinicians, it takes place in a complex clinical setting and requires a high level of interaction between the PRO solution and its context. The MRC framework for developing and evaluating complex interventions consists of four iterative phases: development, feasibility, evaluation, and implementation [17, 20], and it emphasizes that repeating phases is preferable if challenges remain unsolved. Therefore, attention focuses not only on designing the intervention but also on the conditions and resources needed for its successful implementation [17]. This framework stresses the iterative process through different phases, which is pivotal in the development, implementation, and maintenance of PRO solutions in clinical practice. Furthermore, existing PRO-specific guidelines are important to incorporate in the implementation of PROMs in clinical practice. The PROTEUS-Practice Guide offers guidance on designing, implementing, and managing PRO systems and related data in clinical care for healthcare providers [18]. The PROTEUS Guide enhances various key aspects essential for the successful integration of PROMs into clinical practice and components from this guideline have inspired part of the content in our implementation model.

In summary, no single framework addresses all aspects of development, implementation, and maintenance of PRO solutions in clinical practice. Given the dynamic nature of clinical environments, PRO solutions are inherently evolving and benefit from continuous monitoring, evaluation, and adaptation to ensure their long-term sustainability and relevance. This paper presents the AmbuFlex model as a structured approach for developing, implementing, and maintaining PRO solutions in clinical practice.

## The AmbuFlex model

**Fig. 1:**
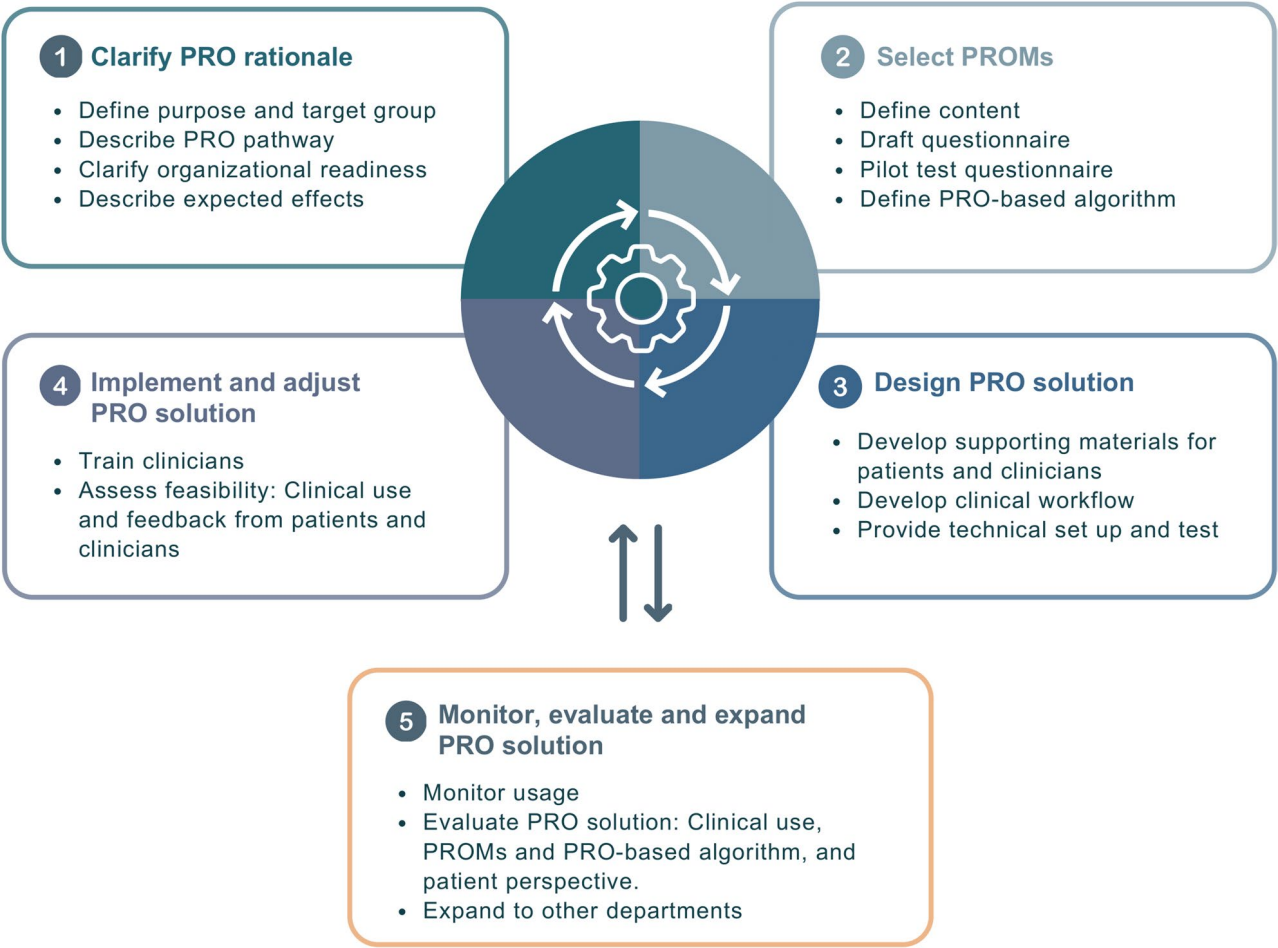
The AmbuFlex model

The AmbuFlex model comprises five steps: (1) Clarify PRO rationale, (2) Select PROMs, (3) Design PRO solution, (4) Implement and adjust PRO solution, and (5) Monitor, evaluate and expand PRO solution. The first three steps describe the development of a new PRO solution before the implementation in step 4, and the final step 5 outlines the maintenance of PRO solutions in clinical practice. Figure 1 shows an overview of the AmbuFlex model, illustrating the different steps involved in developing, implementing, and maintaining PRO solutions in clinical practice. The iterative implementation process in the AmbuFlex model is coordinated by a PRO facilitator in close cooperation with relevant stakeholders. Table 1 shows the barriers and facilitators for developing, implementing, and maintaining PRO solutions in clinical practice. We identified these factors through discussions with patients and clinicians, and from 15 years of experience using the AmbuFlex model. The table’s elements guide our specific actions during implementation. For example, involving relevant stakeholders is essential to ensure clinical value and relevance. Success depends on addressing these factors throughout the implementation process.

**Table 1:** Barriers and facilitators across phases of the AmbuFlex model

Phases	Subthemes	Barriers	Facilitators
Clarify PRO rationale	Utility of PRO and clinical engagement	Ambiguity or disagreement about the purpose Unclear patient pathway and PRO contribution Challenges establishing a well-functioning working group Limited clinician commitment Lack of peer department endorsement	Clearly defined purpose from the outset Clear perceived value Initiative originating within the clinic Multidisciplinary working group Engaged and inquisitive clinicians Prior departmental PRO experience Aligned expectations for process
	Leadership and resources	Top-down mandate for PRO implementation Limited time and resources in the clinic Unmet expectations of the PRO system	Strong leadership support Proactive identification of barriers
Select PROMs	Content and methods	Unclear or disagreement about the purpose of the questionnaire and/or colour coding Disagreement about questionnaire content and/or colour coding Preference for validated scales not well-suited to clinical practice Difficulty obtaining questionnaire license PROM unavailable in Danish PROM unavailable in other languages	Shared understanding of questionnaire purpose and colour coding Examples from other PRO implementations provide inspiration Preference for validated scales that are well-suited to clinical practice
Design PRO solution	Working group process	Limited time and resources in the clinic Turnover in the working group Absence of decision-makers in the working group Lengthy development of patient information	Adequate time and resources for meetings Engaged clinicians Perceived progress in development
	Workflow and technology	Complex or inconsistent clinical workflows Disruption to clinic workflows Technical barriers and limited integration Heterogeneous patient population with diverse needs, placing high demands on the PRO system	Well-defined clinical workflows Clinic involvement in clinical workflow mapping Implementation aligned with clinic operations
Implement and adjust PRO solution	Training and application	Limited time for patient enrolment Low patient volume limiting learning Failure to review patient responses No designated responsible clinician	Dedicated time for training and leadership support Training scheduled close to launch Clinicians recognise value and impact Strong clinical champions Close follow-up and drop-in support from the PRO facilitator Positive patient feedback
	Technical system	IT challenges or system misconfiguration Difficulty troubleshooting or identifying non-respondents across digital systems	User-friendly and agile PRO system Rapid early iterations Rapid access to technical support
Monitor, evaluate and expand PRO solution	Sustained use	High workload and competing priorities Low patient volume reduces visibility Declining clinician interest over time	Sustained leadership prioritisation Clinicians recognising value and impact Clinically relevant questionnaire
	Organisational and clinical changes	New clinical guidelines Changes in workflows or patient pathways Cross-department disagreement about content Delayed PRO system features	Workflow adjustments as needed Clear and simple clinical workflows Sharing experiences in internal networks
	Scale-up and maintenance	Loss of key personnel Insufficient communication about PRO purpose and use Influence of negative anecdotes Reduced ownership during scale‑up	Establishing a maintenance working group Engaged clinicians and dedicated champions Leadership communicating purpose of the PRO solution Continued follow-up by the PRO facilitator

### Step 1: Clarify PRO rationale

This step concerns the establishment of a common understanding of the PRO solution. The process is usually initiated by a hospital department that contacts the AmbuFlex team requesting help to assess whether using a PRO solution in their patient group will help solve one or more of the challenges they encounter in clinical practice. The first step is to clarify whether using PROMs is the right solution and whether PROMs can support their needs in this specific department and similar departments. A PRO facilitator plans the process, involves relevant stakeholders, and facilitates meetings. A working group is established, including relevant stakeholders, such as clinicians and allied professionals, secretaries, leaders, and the PRO facilitator. External clinical experts working within the same clinical field in other Danish hospitals are involved to ensure the development of a sustainable PRO solution, which can be expanded to other similar departments. The PRO facilitator conducts participant observations in the clinic to understand the clinical context and to explore the patients’ perspectives. The working group determines the purpose, the target group, the PRO pathway, organizational readiness, and the expected goals.

#### Define purpose and target group

The foundation for developing a sustainable PRO solution is to understand the rationale behind using PROMs in a clinical context. How PROMs are planned to be used in a specific setting, for what purpose, and for whom? The process of defining a specific purpose in collaboration with the working group is to clarify the specific reasons for collecting PROMs, how to use PROMs, and how PROMs potentially facilitate improvement or optimization of the clinical pathway for patients. However, a PRO solution might have multiple purposes, benefiting patients, clinicians, and the healthcare system. For example, PROMs can support patient-centered communication, identify problems (screening), monitor treatment effects, and create flexible and individualized clinical pathways or treatment plans for patients. The target population is defined, including any specific in- or exclusion criteria, such as linguistic skills or cognitive difficulties.

#### Describe PRO pathway

The PRO pathway describes how PROMs should be integrated into an existing clinical pathway. Designing a pathway that captures PROMs requires an evaluation of the existing patient pathway to be able to identify areas that require modification to facilitate PROM integration. How PROMs are integrated into the existing patient pathway depends on the purpose of using PROMs in that specific clinical context and on the existing pathway in the department for the selected target group. The following questions could be relevant to discuss with the working group: (1) How does the existing workflow function? (2) How do PROMs fit into the process? (3) Is there a need to combine PROMs with other clinical data, such as blood tests? (4) When should patients respond? (5) Which staff groups should manage incoming PRO responses? and (6) Should any existing workflows be de-implemented?

#### Clarify organizational readiness

To prevent potential barriers later in the implementation process, it is important to establish the department’s readiness to implement a complex intervention [21]. If major challenges are identified, these must be considered before further development of a PRO solution takes place. Crucial organizational factors to consider include staff resources, leadership support, readiness for change, logistics challenges [22], , and endorsement by external clinical experts from other similar departments. Barriers among patients and clinicians could also be relevant to consider at this stage of the process [18]. Potential barriers among patients could be problems related to completing PROMs due to physical or cognitive impairment, language issues, and low literacy and digital competencies [18, 23–26]. Moreover, barriers among the clinicians could be disruption of workflows, time and resource constraints, and lack of knowledge of how to interpret and discuss PROMs with patients [27–30]. To address the identified potential barriers a co-creation approach, involving and engaging patients and clinicians in the development of the PRO solution, is essential to achieve successful implementation in practice.

#### Describe expected effects

The expected effects must align with the overall purpose of using the PRO solution and how it is used in a specific clinical context. Since a PRO solution is considered a complex intervention, both proximal and distal outcomes are relevant for evaluation [17]. The outcomes can be divided into three categories: (1) Process outcomes evaluating how the PRO solution is used (proximal effects), e.g., the quality of patient-clinician communication, problem identification, treatment adjustments, response rate and adherence, (2) Patient health outcomes evaluating the distal effect of the PRO solution, e.g., symptom burden, health-related quality of life, and survival, and (3) Health service outcomes evaluating the distal effect of the PRO solution, e.g., satisfaction and use of healthcare services. Expected effects and how and when they are going to be measured should be discussed during this phase to ensure the development of a sustainable PRO solution aiming to bring value to patients, clinicians and the healthcare system.

### Step 2: Select PROMs

The PRO facilitator works closely with patients and clinicians in this step to develop a clinically relevant questionnaire. The process is interactive and iterative, involving defining questionnaire content, drafting and pilot testing the questionnaire, and defining the PRO-based algorithm.

#### Define content

The first step is to define which aspects and levels of health status outcomes should be measured in the target population. This involves identifying clinically relevant aspects, such as disease-related symptoms, physical, mental, or social functioning, and overall health-related quality of life, aligned with the aim of using PROMs in the target population. The PRO facilitator collects input to the content and constructs to be included in the questionnaire, both from clinicians during workgroup meetings and from patients through participant observation in the department.

#### Draft questionnaire

Following content definition, a systematic literature search aims to identify established generic and disease-specific PRO instruments covering the clinically relevant aspects to be measured. If no established PRO instruments are identified, single items are developed. The phrasing of the items follows recommended guidelines, such as unambiguous wording, only to include one dimension, no negations, and exhaustive response categories [31]. Standard phrasing is inspired by established single items, e.g., from the Symptom Checklist 92 (SCL-92) and the European Organization of Research and Treatment in Cancer (EORTC) item library. Subsequently, a questionnaire draft is prepared covering all clinically relevant aspects identified by the clinicians and the patients. The questionnaire draft will often contain both established generic and disease-specific instruments along with self-developed questions. The draft is discussed and adjusted by PRO facilitators and researchers from AmbuFlex and clinicians at workgroup meetings. In addition, the draft is also assessed by external clinical experts from other departments. When agreement has been achieved, the questionnaire is considered ready to be tested in a sample of the target population.

#### Pilot test questionnaire

The questionnaire draft is pre-tested among 7 to 8 patients in the target population using the cognitive interview techniques “think aloud” and “cognitive probing” [32]. The pilot test aims to evaluate face and content validity assessing relevance, comprehensiveness and comprehensibility [33]. Two PRO facilitators participate in the test, in which one person is responsible for conducting the interview, and the other person conducts observations and takes notes during the interview [34]. Following each interview, data are analysed. The results from the interviews and issues raised by the patients are discussed with the clinicians at working group meetings. The aim is to consider the necessary changes, e.g., whether it is necessary to rephrase, delete, or add items before the final version of the questionnaire becomes available. Modifications are limited to self-developed single items. No modifications are made to established, validated PROMs. Following this discussion, a draft of the questionnaire is finalized. PRO instruments used in clinical practice often consist of multiple single-domain items designed to inform patient management. Their validation therefore extends beyond traditional psychometric testing and requires a clinimetric approach, focusing on clinically meaningful outcomes, intended use, and performance in real-world care. Importantly, validation in this setting concerns not only measurement properties in isolation, but the extent to which the PROM is valid within the specific clinical context in which it is applied, ensuring relevance, interpretability, and fit for purpose in routine practice [35, 36].

#### Define PRO-based algorithm

Based on the final draft of the questionnaire, clinicians in the working group assign the response categories for each item into green, yellow, or red color-codes. The allocation within the color-coded algorithm is primarily grounded in clinical expertise and, where available, established threshold or clinical guidelines. Thresholds and decision rules are defined based on what clinicians consider clinically important to act upon, aligned with the overall purpose of the PRO solution. The algorithms serve different purposes and to some extent, make decisions that should align with a clinician’s assessment of the individual patient [7, 8]. The algorithm thus reflects predefined criteria for response, ensuring that patient-reported data trigger actions consistent with clinical priorities and standards of care. Often, the aim of the algorithm follows a ‘red flag’ approach. In this case, the assigned color codes of all relevant response categories are used to generate an overall color code algorithm for the patient’s questionnaire response, which can be green, yellow, or red. For example, if a patient has a red response to a question, the entire questionnaire response is marked as red, indicating that the patient needs clinical attention. A yellow color indicates that the patient may need clinical attention, and a green color indicates that the patient does not need clinical attention [11, 37]. The PRO-based algorithm threshold aims to balance false negative and false positive responses. It aims to prevent false negatives, ensuring that no patients requiring clinical attention are assigned a green color code. However, a high number of false positives can burden clinicians, as they must assess red color-coded responses from patients who do not require clinical attention. The questionnaire often includes a question about the patient’s perception of their need for contact, recognizing that there may be additional reasons for clinical contact beyond those covered in the questionnaire. Not all questions are necessarily assigned a color code, and not all solutions use an overall color code for the response. PRO solutions without an overall color code are used exclusively to support communication during face-to-face consultations. Individual items indicate symptom severity, and the dialogue focuses on those with the highest severity.

### Step 3: Design PRO solution

At this stage, supporting materials for patients and clinicians, and a detailed plan regarding how to incorporate PROs into the clinical workflow are developed. Moreover, the PRO facilitator conducts the technical configuration of the PRO solution in the AmbuFlex system. Needs for adjustment are discussed with members of the workgroup.

#### Develop supporting materials for patients and clinicians

During meetings, the workgroup considers the development of tailored materials for patients and clinicians. The supporting materials are co-designed together with end-users. Examples of materials provided to patients could include instructions about when and how to complete the questionnaire, feedback about a specific questionnaire response, guidance on how to access their previous questionnaire responses, and information about the questionnaire’s content. The working group considers the need for developing materials for their colleagues, such as a clear description of the workflow and action plans for yellow/red responses, e.g., should a specific answer indicate a recommended action, for example, referral to a psychologist. The materials are available for all clinicians via an electronic documentation system. Additionally, clinicians receive a manual outlining the functionalities of the AmbuFlex software, such as instructions for logging into the system, referring patients, and managing incoming PRO responses.

#### Develop clinical workflow

During workgroup meetings, members develop a new clinical workflow, considering new assignments, responsibilities, and the timing and location for completing tasks. These considerations are discussed for key assignments, including how to enroll patients into the PRO solution, collect the PRO responses from patients, and how to review incoming PRO responses. At each step, a list of questions can be discussed in the workgroup before finalizing the workflow (Table 2).

**Table 2:** Overview of assignments and questions in the development of a clinical PRO workflow

Assignment	Relevant questions
The patients need to be enrolled in a specific PRO solution	How will patients be informed and enrolled in the PRO solution? Who is responsible for this assignment? Who will be enrolled, e.g., all patients or a specific subgroup?
The patients must respond to the questionnaire	How often and when should patients complete questionnaires? Should questionnaire timing align with collecting other types of clinical data, e.g., a blood sample? How will patients receive the questionnaire? How will patients be reminded and supported? How will patients respond to the questionnaire (mode of administration), e.g. web, by proxy? Where will the questionnaire be collected, e.g., at home/remote or in-clinic?
The incoming PRO responses need to be tracked, reviewed and actions must be documented	How will non-responders and missing data be managed? When, where and to whom will PRO responses be presented, e.g., patients, clinicians, or both? Who from the clinical team will be responsible for reviewing incoming PRO responses? Should reviews and actions of incoming PRO responses be based on clinical guidelines? How will the actions be documented?

#### Provide technical set up and test

The AmbuFlex system’s technical configuration consists of three generic elements, including PRO collection, a PRO-based algorithm, and a PRO-based graphical overview [8]. The PRO facilitator is responsible for the technical configuration of the PRO solution in cooperation with the IT team in AmbuFlex. The technical setup of the PRO solution is determined by its intended purpose and how it will be utilized within a specific clinical context. The first part of the technical configuration consists of a questionnaire setup followed by the associated PRO-based algorithm setup. The second part includes technical configuration of (1) general settings, e.g., whether the system will send feedback letters or self-management guidance to patients and whether patients will be automatically withdrawn after a specific period, (2) settings for clinicians, e.g., how clinicians will handle incoming PRO responses and what kind of patient lists that will be available, and (3) settings for the website where patients access the questionnaire, e.g., a website headline and information text. Patients can access their previous PRO responses via the patient portal. The third part of the technical configuration consists of the setup of the PRO-based graphical overview shown to the clinicians within the electronic health record. Finally, before feasibility testing of the PRO solution in clinical practice, two members of the AmbuFlex team individually conduct a thorough safety review of the content and technical configuration. Following the technical configuration, clinicians provide input on the content of the graphical PRO overview and the functionalities of the PRO solution within the electronic health record. During this process, the PRO solution is adjusted before formal roll-out in clinical practice.

### Step 4: Implement and adjust PRO solution

At this stage, the department begins using the PRO-solution in clinical practice, and the PRO facilitator conducts training sessions with the involved clinicians. Moreover, the PRO facilitator is considering whether to arrange drop-in visits at the department to clarify potential questions and safety issues raised by the clinicians. The number of drop-in visits depends on the department’s prior experience of using PRO solutions, the time and staff resources in the department, and potential challenges that arise. The PRO facilitator continuously monitors the usage of the PRO solution to identify potential challenges. In addition, AmbuFlex offers daily technical telephone support to patients and clinicians. This support can help identify pivotal IT system flaws and assess the need for extra support in specific patient groups.

#### Train clinicians

The PRO facilitator and workgroup members from the department arrange training sessions for clinicians related to different aspects of using PROMs in clinical practice, e.g., (1) The overall purpose of the PRO solution and how the solution will affect the clinical workflow, (2) Patient-centered communication and the importance of using PROMs in the clinical encounter, and (3) AmbuFlex software functionalities, e.g., referring patients and managing incoming PRO responses. The training sessions are not standardized at fixed time points but are arranged based on the context and the department’s preferences. Training modalities include individual peer-to-peer training or group sessions. The aim is to offer training to all clinicians involved in using the PRO solution in clinical practice, including physicians, nurses, and allied professionals.

#### Assess feasibility

The usability and feasibility of the PRO solution are assessed approximately three months after implementation to capture real-world workflow. During this three-month period, the PRO facilitator monitors usage data through the AmbuFlex database, tracking (1) the number of patients enrolled in the PRO solution, (2) questionnaire completion rates, (3) the distribution of the PRO-based algorithm, and (4) whether clinicians review the incoming PRO responses. In addition, the PRO facilitator conducts drop-in visits and collects ongoing feedback from patients and clinicians based on informal dialogues and issues raised by users who have contacted the AmbuFlex support team. Any safety-related issues trigger an immediate review and, if necessary, adjustment to the PRO solution (e.g., technical configuration, patient information, or the PRO-based algorithm) is implemented during or after this monitoring period.

### Step 5: Monitor, evaluate, and expand PRO solution

Due to the dynamic nature of clinical practice, a PRO solution cannot remain static. Thus, the PRO facilitator continuously monitors the usage of the PRO solution and considers whether a drop-in visit at the department is necessary if problems occur. Furthermore, the PRO facilitator arranges a joint meeting to discuss the need to adjust the PRO solution with all involved departments.

#### Monitor usage

PRO data from the AmbuFlex database are integrated with other data sources, including electronic patient records through a centralized data warehouse in the Central Denmark Region. This enables monitoring and analysis of PRO data in relation to the patient’s treatment trajectory and regularly monitoring of usage data, supporting quality improvement of the PRO solutions and their clinical application. In addition, inquiries from the telephone support are monitored to assess the volume of patient inquiries related to the PRO solution, as this may indicate potential implementation challenges. If necessary, drop-in visits at the department are arranged.

#### Evaluate PRO solution

The PRO solutions are evaluated to assess their alignment with user requirements, organizational efficiency, and the perceived benefits for patients and clinicians using the PRO solution. Our evaluation approach is process-oriented and pragmatic, drawing inspiration from the formative evaluation theory [38]. This theory emphasizes the importance of enhancing knowledge about interventions to enable improvements. Additionally, we are inspired by the “Model for Assessment of Telemedicine” (MAST), developed for evaluating the effect of telemedicine interventions [39]. The MAST model has been used to define three domains that form the basis of our evaluations of PRO solutions, including (1) clinical use, (2) the PROMs and PRO-based algorithm, and (3) the patient perspective.

#### Clinical use

Regular monitoring of the patients’ and clinicians’ usage of the PRO solution plays a crucial role in the evaluation of the PRO solutions as it helps to ensure a sustainable and high-quality PRO solution. The monitoring consists of data regarding the quantity of what is delivered and how it is used and covers the following domains: reach, fidelity, and dose [40] (Table 3). In addition, clinical engagement is assessed by collecting feedback from clinicians with experience in using the PRO solution in clinical practice (Table 3).

**Table 3:** Key domains in the monitoring of PRO solution activities

Domain	Relevant questions	Method	Data source
Reach	Who receives the PRO solution? Is the target population representative of the whole patient population?	Number of patients enrolled Number of patients at the department with similar diagnosis	Hospital Business Intelligence Register AmbuFlex database
Dose	To what extent have the patients received and engaged with the PRO solution?	Number of distributed questionnaires Number of reminders Number of questionnaire responses Number of item responses Time spent completing the questionnaire Number of non-responders Distribution of the PRO-based algorithm (green, yellow, red color-codes)	AmbuFlex database
Fidelity	Is the PRO solution delivered as intended? Do the clinicians incorporate the patients’ PRO responses in the consultation?	Number of clinics and clinicians using the PRO solution Do the clinicians review the PRO overview in the electronic health record? Clinician assessments of patients’ PRO responses, e.g., contact/no contact	AmbuFlex database
Clinical engagement	How do the clinicians perceive the PRO solution in clinical practice?	Informal conversations or semi-structured interviews with clinicians	Involved clinicians with experience using the PRO solution

#### PROMs and PRO-based algorithm

The selected PROMs and PRO-based algorithm are central elements in the PRO solutions. However, due to the dynamic nature of clinical practice, the questionnaire content and algorithm may need to be evaluated and adjusted. The PROMs and PRO-based algorithm are evaluated by PRO facilitators and clinical experts from departments using the PRO solution. Descriptive data from the AmbuFlex database, such as item distribution, missing data, and algorithm distribution are used. In addition, a systematic literature search is conducted to identify if potential new relevant PRO instruments emerged since the development of the PRO solution.

PRO solutions that incorporate PRO-based algorithms to support patient management or clinical triage are classified as medical devices under Regulation 2017/745 of the European Parliament and Council (MDR), and complies with the MDR’s general safety and performance requirements [41]. For example, PRO-based algorithms aiming to triage patients who need clinical attention undergoes evaluation to ensure its quality, focusing on “green” algorithm outcomes, where in some PRO solutions, the patient is not contacted at all. Algorithm outcomes are linked with patient data from electronic health records and analyzed to identify potential critical incidents. In this way, monitoring and documentation of patient safety is an integrated part of the AmbuFlex model.

#### The patient perspective

The PRO solution affects the included patients by introducing a new patient pathway. For example, it may change the nature and frequency of their interactions with healthcare professionals and places demands on patients regarding their understanding of the disease and technical competencies. At the same time, patient satisfaction is crucial for the PRO solution to be perceived as valuable. Data are collected through semi-structured interviews with 5–10 enrolled patients. The interviews are conducted using a structured guide that covers the relevance, content, and comprehensibility of the questionnaire. The guide also explores patients’ experiences of completing the questionnaire, how their responses are used in clinician interactions, and how the questionnaire supports the management of their illness. The interviews are conducted by a PRO facilitator or a researcher from AmbuFlex, then transcribed and analyzed thematically [42].

#### Expand to other departments

Once the PRO solution has been tested and refined through clinical use, other departments may implement it. Until now, the expansion of PRO solutions to other departments has been successfully driven by clinical demand rather than a top-down approach. We believe this is partly due to the AmbuFlex model, as we involve clinicians from other hospitals in the development process. In this way, clinicians not only help enhance the quality of the solution but also raise awareness and encourage broader adoption.

The AmbuFlex team facilitates the expansion within the Central Denmark Region, working with a local implementation facilitator at each hospital. Other Danish regions can also implement the existing PRO solution free of charge.

## Discussion

Over the years, AmbuFlex has developed and gained extensive experience with a generic and adaptable model to successfully develop, implement, and maintain PRO solutions in clinical practice. The AmbuFlex model consists of five steps: (1) Clarify PRO rationale, (2) Select PROMs, (3) Design PRO solution, (4) Implement and adjust PRO solutions, and (5) Monitor, evaluate and expand PRO solution. Each step is pivotal to consider during the iterative implementation process. From 2011 to 2026, a total of 90 specific PRO solutions have been developed and implemented at hospital departments in two Danish Regions based on the AmbuFlex model. Several case studies based on the model have been published [43–46].

Nilsen describes different types of theories, models, and frameworks in implementation science [47]. Overall, three types of theoretical approaches are described: (1) process models guiding the process of translating research into practice, (2) determinant frameworks, classic theories, and implementation theories understanding and/or explaining what influences implementation outcomes, and (3) evaluation frameworks evaluating implementation [47]. The AmbuFlex model fits into the process model categorization, as our model has a knowledge-to-action approach facilitating implementation by offering practical guidance in the planning and execution of implementation strategies [47]. The AmbuFlex model includes steps that need to be considered to cause a change in clinical practice when developing, implementing, and maintaining a PRO solution. It should be noted that, although the steps in the AmbuFlex model are presented stepwise, the process is iterative. There is often a need to move back and forth between the steps, depending on the context in which the PRO solution is implemented. The time from step 1 to step 4 ranges from 6 to 9 months, however, these steps may take more or less time depending on factors such as the selected PROMs, the complexity of changes in the clinical workflow, the resources available in the clinical department, and the workload in the AmbuFlex team. The last step in the model continues unless a decision is made to de-implement a PRO solution. This phase persists due to the dynamic nature of clinical practice and the ongoing need to monitor, evaluate, and adjust the PRO solution to ensure its long-term sustainability. When maintaining PRO solutions, it is often necessary to revisit earlier steps to ensure the PRO solution continues to align with the clinical context.

Like other models described in the literature [47], the AmbuFlex model is based on practical experiences of developing, implementing, and maintaining PRO solutions drawing inspiration from existing guidelines and frameworks, such as the PROTEUS Guide to Implementing PROs in Clinical Practice [18], the CFIR Framework for Implementation Research [16], and the MRC framework of development and evaluation of complex interventions [17]. This combination is essential to the AmbuFlex model as it acknowledges the unique AmbuFlex approach of considering the complexity of implementing PROMs in clinical practice.

Recently, a case study described the Amsterdam PROM Pathway [48]. The Amsterdam PROM pathway is a practical guideline for implementing PROMs in clinical care across diverse patient populations. The Amsterdam PROM Pathway consists of four phases: registration, design, preparation, and implementation [48]. This guideline shares some similarities with the AmbuFlex model, for example, the Amsterdam model employs a systematic yet flexible bottom-up implementation approach to accommodate diverse patient groups and workflows. Also, the model is based on over 15 years of real-life experiences using the KLIK PROM portal in more than 40 Dutch hospitals [48]. However, unlike the AmbuFlex model, the Amsterdam model was developed to support the execution of a national PROM implementation policy. This policy consists of eight recommendations, including determine objective, select PROs, select PROMs, test the PROM, define the indicator, test the indicator, use the PROM, and maintenance and evaluation [49]. The AmbuFlex model is used to assist local hospital departments in conducting the stepwise PROM implementation process and to describe the content of each step within this process, including ensuring maintenance of implemented PRO solutions. Although being part of a publicly funded organization provides advantages, such as established infrastructure and institutional support, it is not a prerequisite for implementing the AmbuFlex model. Implementation is primarily driven by the clinical need and the value the PRO solution provides for patients, clinicians, and the health system. Consequently, the model’s principles can be adapted to other organizational and funding contexts.

### Future directions and considerations

Over the last decade, we have witnessed a rapid development of digital tools in the healthcare system. ‘Digital health’ is a broad concept covering terms such as electronic health (eHealth), mobile health (mHealth), and telemedicine. All terms involve the use of information and communication technology (ICT) in support of health [50]. Similarly, the use of electronic patient-reported outcomes (ePRO) has become more widespread in capturing patient-reported data in the healthcare system [51]. Several advantages of using ePRO data collection have been pointed out, such as, immediately accessible data, automatic scoring of data, and more accurate and complete data [52]. AmbuFlex has transitioned from a mixed-mode approach (web and paper) for PRO administration to using ePRO [8, 53]. Over the last few years, the use of ICT systems and new ICT opportunities has continued to evolve in the healthcare system. For example, since 2016 a digital care guide (Emento) has been utilized in several hospitals in the Central Denmark Region [54]. This digital care guide is a generic two-way ICT system/APP that provides patients with information and assignments during treatment and follow-up [54]. Additionally, patients can chat with their healthcare provider if relevant questions arise during their care pathway. If a PRO solution is implemented in a department using the digital care guide, we often recommend integrating the PRO questionnaire into the Emento APP and assigning it as a task for patients. This integration can simplify the overall use of PROMs and supports timely completion. Furthermore, for patients with multimorbidity, parallel care pathways with numerous appointments and tasks often occur. As a result, integrating various ICT systems and creating a comprehensive and user-friendly overview for patients is challenging but remains a critical goal for the future of the digital healthcare system.

The adoption of digital tools in healthcare increases the risk of excluding patient groups with low literacy and digital competencies When using PROMs, it is important to consider respondent burden to prevent low completion rates, missing PRO data, or participant withdrawal [55]. Based on AmbuFlex’s 15 years of experience, we have observed that only a distinct group of patients participates in the PRO solutions [10, 56, 57], with participation rates ranging from 35% in rheumatoid arthritis [58] to 77% in inflammatory bowel disease [57]. Studies have shown that patients with a higher socioeconomic background and a high level of health literacy are more likely to participate in the PRO solutions, while vulnerable patients tend to have lower participation rates [56, 58, 59]. Nevertheless, incorporating PRO data into healthcare has significant potential to improve outcomes for vulnerable patients and address health disparities [60, 61]. Therefore, a future goal is to prioritize digital inclusion and adopt a more ambitious approach to accommodate patients with diverse needs. Rather than designing PRO solutions tailored to standardized care pathways, there is a need for flexible, fit-for-purpose PRO solutions that accommodate individual needs and varying levels of digital competencies.

## Conclusion

In conclusion, the AmbuFlex model is a flexible, generic and adaptable model applicable to developing, implementing, and maintaining various PRO solutions in the healthcare system regardless of purpose, target group, clinical context, and stakeholders involved. The successful implementation of PRO solutions depends on early, context-specific identification of their value to patients, clinicians, and the organization, as well as strong leadership commitment and prioritization. These factors facilitate the allocation of adequate resources, promote clinical engagement, and support the co-design of clear PRO purposes, PRO pathways, and clinical workflows. From 2011 to 2026, a total of 90 specific PRO solutions across diverse patient populations have been developed, implemented, and maintained based on the AmbuFlex model. Thus, the model is a practical and adaptable guideline for developing, implementing, and maintaining sustainable PRO solutions in the healthcare system.

## Data Availability

Not applicable.

## Abbreviations

PRO: Patient-reported outcome
PROM: Patient-reported outcome measure
CFIR: Consolidated Framework for Implementation Research
MRC: Medical Research Council
SCL-92: Symptom Checklist 92
EORTC: European Organization of Research and Treatment in Cancer
MAST: Model for Assessment of Telemedicine
eHealth: electronic health
mHealth: mobile health
ICT: Information and communication technology
ePRO: electronic patient-reported outcomes
